# Supplementary material for: Observation of high-order quantum Pancharatnam-Berry phase with structured photons
Source: Fundam Res. 2024 Dec 3;6(3):1417–22. doi: 10.1016/j.fmre.2024.11.016 (PMC13247495; doi:10.1016/j.fmre.2024.11.016)
Supplement: Supplementary Data S1 — Supplementary Raw Research Data. This is open data under the CC BY license http://creativecommons.org/licenses/by/4.0/ [file mmc1.pdf]

Supplemental Material

Observation of High-Order Quantum Pancharatnam-Berry Phase with Structured Photons

Shuang-Yin Huang,[1,2,*] He Jiang,[1,2,*] Zhi-Cheng Ren,[1,2,†] Zi-Mo Cheng,[1,2] Wen-Zheng Zhu,[1,2] Jing Gao,[1,2] Chang Liu,[3] Xi-Lin Wang,[1,2,4,5,‡] and Hui-Tian Wang[1,2,6]

[1]*National Laboratory of Solid State Microstructures and School of Physics, Nanjing University, Nanjing 210093, China*

[2]*Collaborative Innovation Center of Advanced Microstructures, Nanjing University, Nanjing 210093, China*

[3]*Shandong Institute of Quantum Science and Technology Co. Ltd, Jinan, 250101, China*

[4]*Hefei National Laboratory, Hefei 230088, China*

[5]*Jiangsu Physical Science Research Center, Nanjing 210093, China*

[6]*Collaborative Innovation Center of Extreme Optics, Shanxi University, Taiyuan 030006, China*

*These authors contributed equally to this work.

†Corresponding author.
zcren@nju.edu.cn

‡Corresponding author.
xilinwang@nju.edu.cn

I. High-order Pancharatnam-Berry phase

Berry showed that a state $|\psi(\mathbf{R})\rangle$ undergoing a cyclic transformation upon return to its initial state over a circuit C with respect to its parameters $\mathbf{R}$ will acquire an additional phase given by

$$\begin{aligned}\mathbf{A} &= j\langle\psi(\mathbf{R}) \mid \nabla_{\mathbf{R}}\psi(\mathbf{R})\rangle,\\ \mathbf{V}(\mathbf{R}) &= \nabla_{\mathbf{R}} \times \mathbf{A},\\ \gamma_C &= -\iint_C d\mathbf{S}\cdot\mathbf{V}(\mathbf{R}),\end{aligned} \tag{S1}$$

where $\mathbf{A}$ is the associated "vector potential" referred to as the Berry connection, $\mathbf{V}$ plays the role of a "magnetic field" in the parameter space referred to as the Berry curvature and ∇ is a gradient with respect to the parameters. γ_C is the additional geometric phase. In the parameter space of the high-order Poincaré sphere (PS) with spherical coordinates (β, φ), the arbitrary state $|\psi(\beta, \varphi)\rangle$ on the sphere can be described by [36]

$$|\psi(\beta, \varphi)\rangle = \cos\left(\frac{\beta}{2}\right)|R, m\rangle\, e^{j\varphi/2} + \sin\left(\frac{\beta}{2}\right)|L, -m\rangle\, e^{-j\varphi/2}, \tag{S2}$$

where $|\pm m\rangle$ is the OAM eigenstate with the vortex phase $\exp(\pm jm\phi)$, and $|R\rangle$ and $|L\rangle$ represent the right and left circular polarization with spin angular momentum (SAM) $\sigma = \pm 1$. Under the parameter space of spherical coordinates (β, φ), the arbitrary state $|\psi(\beta, \varphi)\rangle$ on the sphere can be rewritten as

$$|\psi(\beta, \varphi)\rangle = \cos\left(\frac{\beta}{2}\right)|R\rangle\, e^{jm\varphi/2}e^{j\sigma\varphi/2} + \sin\left(\frac{\beta}{2}\right)|L\rangle\, e^{-jm\varphi/2}e^{-j\sigma\varphi/2} \tag{S3}$$

where m is independent of the polarization. The vortex phase $\exp(\pm jm\phi)$ will become $\exp(\pm jm\varphi/2)$. The factor of 1/2 is a consequence of exploiting the 2→1 homomorphism between the physical SU(2) space of the light beam and the topological SO(3) space of the high-order PS. The components of the Berry connection can be obtained as [37]

$$\begin{aligned}\mathbf{A}_\rho &= 0,\\ \mathbf{A}_\beta &= 0,\\ \mathbf{A}_\varphi &= -\frac{(m+\sigma)\cos\beta}{2\rho\sin\beta}.\end{aligned} \tag{S4}$$

The Berry curvature is given by

$$\begin{aligned}\mathbf{V}_\rho &= -\frac{m+\sigma}{2\rho^2},\\ \mathbf{V}_\beta &= 0,\\ \mathbf{V}_\varphi &= 0,\end{aligned} \tag{S5}$$

where the Berry curvature is proportional to the total angular momentum (TAM, $J = m + \sigma$) of light. By $d\mathbf{S} = \rho^2 \sin\beta d\rho d\beta d\varphi$, The resulting geometric phase on the high-order PS is then given

by

$$\gamma_C = -\iint_C d\mathbf{S} \cdot \mathbf{V}(\mathbf{R}) = -(m + \sigma)\Omega/2, \tag{S6}$$

where $\Omega = \iint_C \sin\beta d\beta d\varphi$ is the surface area on the high-order PS enclosed by the circuit C, and it indicates also in fact an opened solid angle of the geodesic surface enclosed by the circuit C on the high-order PS of unit radius. Equation (S6) shows the high-order Pancharatnam-Berry (PB) phase is directly proportional to the TAM of light. For the case of $m = 0$, the high-order PB phase degenerates into the well-known PB phase $\Phi^{SAM} \propto \sigma\Omega/2$. The high-order PS can be seen as a combination of mode sphere and standard PS. The high-order PB phase can be regard as the linear superposition of the geometric phases generated by the evolution of spatial modes and polarization states. So, the geometric phase obtained from a cyclic mode-preserving transformation without the evolution of polarization ($\sigma = 0$) is $\Phi^{OAM} \propto m\Omega/2$.

The PB phase based on the PS can be acquired by a control unit composed of a half-wave plate (HWP) sandwiched between two quarter-wave plates (QWPs), as shown in Fig. 1 of the main text. We set two QWPs at 45° and HWP at θ to acquire the controllable PB phase. The first QWP at 45° converts the horizontal polarization $|H\rangle$ to the left-circular polarization $|L\rangle$ located at the south pole of the PS. The HWP at θ changes continuously into the right-circular polarization $|R\rangle$ located at the north pole along the longitude line of 2θ on the PS, and finally the second QWP at 45° returns to the initial polarization $|H\rangle$. As a whole, the initial polarization $|H\rangle$ on the equator experiences an evolution along the anticlockwise closed trajectory (red solid-line), which surrounds a solid angle of $\Omega = 4\theta$ on the PS, and a PB phase of 2θ will be acquired based on the well-known definition of PB phase. Similarly, when the vertical polarization $|V\rangle$ evolves and finally return into its initial state along the closed trajectory (red dashed line), which also surrounds a solid angle of $\Omega = 4\theta$ on the PS, a PB phase of -2θ will also be acquired due to the clockwise closed trajectory [44]

$$\begin{aligned} &|H\rangle \xrightarrow{\text{QWP}} |L\rangle \xrightarrow{\text{HWP@}\theta} |R\rangle \xrightarrow{\text{QWP}} e^{j2\sigma\theta}|H\rangle, \\ &|V\rangle \xrightarrow{\text{QWP}} |R\rangle \xrightarrow{\text{HWP@}\theta} |L\rangle \xrightarrow{\text{QWP}} e^{-j2\sigma\theta}|V\rangle. \end{aligned} \tag{S7}$$

For the geometric phase based on the OAM mode sphere, we use a q-plate sandwiched between two QWPs to make the $|H, 0\rangle$ photons into $|H, m\rangle$ and the $|V, 0\rangle$ photons into $|V, -m\rangle$, respectively. After passing through the first Dove prism (DP) at $\pi/4m$, the initial state $|H, m\rangle$ at the north pole turns into $|H, -m\rangle$ at the south pole with a initial phase of $\exp(j\pi/2)$. Then, pass through the second DP with rotation angle θ respect to the first one. $|H, -m\rangle$ becomes the initial state $|H, m\rangle$ with the longitude line of 2θ. As a whole, the initial polarization $|H, m\rangle$ experiences an evolution along the anticlockwise closed trajectory (red solid-line), which surrounds a solid angle of $\Omega = 4\theta$ on the mode sphere, a geometric phase of $2m\theta$ can be acquired. Similarly, when the $|V, -m\rangle$ evolves into its initial state along the closed trajectory (red dashed line), which also surrounds a solid angle of $\Omega = 4\theta$ on the mode sphere, a geometric phase of $-2m\theta$ will be acquired, due to the clockwise

closed trajectory

$$\begin{aligned}|H,0\rangle &\xrightarrow{\text{QWP}} |L,0\rangle \xrightarrow{\text{q-plate}} |R,m\rangle \xrightarrow{\text{QWP}} |H,m\rangle \xrightarrow{\text{DP@}\pi/4m} e^{j\pi/2}|H,-m\rangle \xrightarrow{\text{DP@}\theta} je^{j2m\theta}|H,m\rangle \\ &\xrightarrow{\text{QWP}} je^{j2m\theta}|R,m\rangle \xrightarrow{\text{q-plate}} je^{j2m\theta}|L,0\rangle \xrightarrow{\text{QWP}} je^{j2m\theta}|H,0\rangle, \\ |V,0\rangle &\xrightarrow{\text{QWP}} |R,0\rangle \xrightarrow{\text{q-plate}} |L,-m\rangle \xrightarrow{\text{QWP}} |V,-m\rangle \xrightarrow{\text{DP@}\pi/4m} e^{-j\pi/2}|V,m\rangle \xrightarrow{\text{DP@}\theta} -je^{-j2m\theta}|V,-m\rangle \\ &\xrightarrow{\text{QWP}} -je^{-j2m\theta}|L,-m\rangle \xrightarrow{\text{q-plate}} -je^{-j2m\theta}|R,0\rangle \xrightarrow{\text{QWP}} -je^{-j2m\theta}|V,0\rangle.\end{aligned} \tag{S8}$$

Here $|\pm m\rangle$ are the OAM eigenstates with the vortex phase $\exp(\pm jm\phi)$. $|P,m\rangle$ stands for the $|P\rangle$ polarized OAM eigenstate with the vortex phase $\exp(jm\phi)$, where $|P\rangle$ can be $|H\rangle$, $|V\rangle$, $|R\rangle$ or $|L\rangle$, especially $|P,0\rangle$ is the $|P\rangle$ polarized fundamental Gaussian mode with $m=0$.

The high-order PB phase based on high-order PS can be acquired by the same procedure. The first QWP at 45° makes the $|H,0\rangle$ state evolve to the $|L,0\rangle$ one. After passing through the q-plate, the $|L,0\rangle$ becomes into the $|R,m\rangle$ OAM mode at the north pole. The first set of HWP+DP at $\pi/4(m+\sigma)$ transforms the state $|R,m\rangle$ into $|L,-m\rangle$ at the south pole with an initial phase of $\exp(j\pi/2)$ and then the second set of HWP+DP at a rotation angle θ about the first one makes $|L,-m\rangle$ return into the initial state $|R,m\rangle$ at the north pole. Thus the anticlockwise closed trajectory (red solid line) surrounds a solid angle of $\Omega=4\theta$ on the high-order PS and a high-order PB phase of $2(m+\sigma)\theta$ will be acquired for the state $|R,m\rangle$. Similarly, the initial state $|L,-m\rangle$ will also experience a cyclic evolution along the closed trajectory (red dashed line), which also surrounds a solid angle of $\Omega=4\theta$ on the high-order PS, a geometric phase of $-2(m+\sigma)\theta$ will be acquired due to the clockwise closed trajectory. At the end, we can further convert the vector mode into fundamental Gaussian mode by a q-plate and a QWP, and the geometric phase acquired by the evolution of the vector mode is transferred to the relative phase of the $|H\rangle$ and $|V\rangle$ components.

$$\begin{aligned}|H,0\rangle &\xrightarrow{\text{QWP}} |L,0\rangle \xrightarrow{\text{q-plate}} |R,m\rangle \xrightarrow{\text{HWP+DP@}\pi/4(m+\sigma)} e^{j\pi/2}|L,-m\rangle \xrightarrow{\text{HWP+DP@}\theta} je^{j2(m+\sigma)\theta}|R,m\rangle \\ &\xrightarrow{\text{q-plate}} je^{j2(m+\sigma)\theta}|L,0\rangle \xrightarrow{\text{QWP}} je^{j2(m+\sigma)\theta}|H,0\rangle, \\ |V,0\rangle &\xrightarrow{\text{QWP}} |R,0\rangle \xrightarrow{\text{q-plate}} |L,-m\rangle \xrightarrow{\text{HWP+DP@}\pi/4(m+\sigma)} e^{-j\pi/2}|R,m\rangle \xrightarrow{\text{HWP+DP@}\theta} -je^{-j2(m+\sigma)\theta}|L,-m\rangle \\ &\xrightarrow{\text{q-plate}} -je^{-j2(m+\sigma)\theta}|R,0\rangle \xrightarrow{\text{QWP}} -je^{-j2(m+\sigma)\theta}|V,0\rangle.\end{aligned} \tag{S9}$$

II. Preparation and measurement of PB phase for the single photon state

A linearly polarized 390 nm pump beam is incident into type-II PPKTP2 to create a pair of collinear photons via the spontaneous parametric down-conversion (SPDC) process shown in Fig. S1. A polarization beam splitter (PBS) splits $|H\rangle$ and $|V\rangle$ polarized down-converted photons into two single-photon avalanche photodiodes (D1 and D2) as a heralded single photon source.

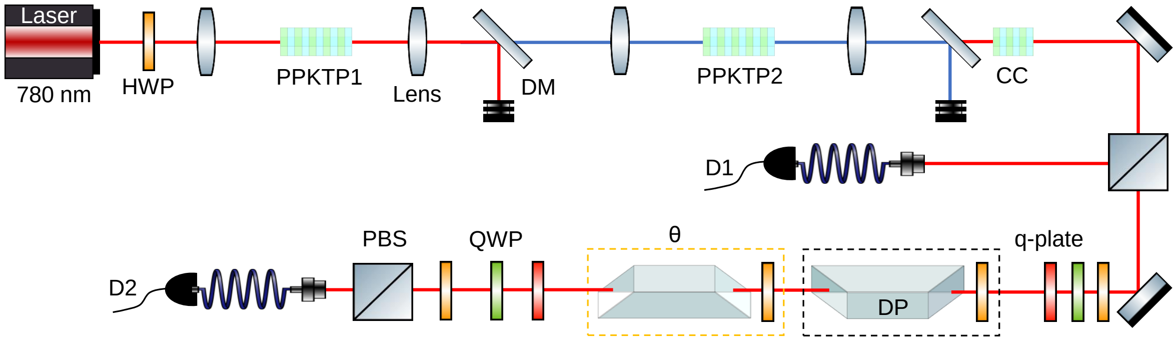

FIG. S1: Experimental setup for measuring the high-order PB phase with the single photon state. A Gaussian beam at λ= 780 nm is doubled to 390 nm by type-II PPKTP1 crystal. A linearly polarized 390 nm beam is incident into type-II PPKTP2 to create a pair of collinear photons via the SPDC process. DM is the dichroic mirror to isolate the pump beam. The extra birefringent crystals CC is used to compensate for the birefringent walk-off effects from the production crystal. A polarization beam splitter (PBS) separates $|H\rangle$ and $|V\rangle$ into two single-photon avalanche photodiodeds (D1 and D2) as a heralded single photon source. Passing through the HWP at 22.5°, photons in $|H\rangle$ will turn into the $|H\rangle + |V\rangle$. The high-order structured photons can be created with QWP and q-plate. The control unit of high-order PB phase is composed of two HWP+DPs. The second q-plate and QWP convert structured photons into fundamental mode. A HWP at 22.5° and a PBS will project photons into the basis of $|D\rangle$. Then, photons are collected by single mode fiber detected with D1 and D2.

Passing through the HWP at 22.5°, photons in $|H\rangle$ will be transformed into $|D\rangle = \frac{1}{\sqrt{2}}(|H\rangle + |V\rangle)$. For the initial state $|\psi_1\rangle$, it undergoes a cyclic transformation to return to its initial state $|\psi_1'\rangle$. A PB phase of $(m+\sigma)\Omega/2$ will be acquired for the $|H\rangle$ and $-(m+\sigma)\Omega/2$ will be acquired for the $|V\rangle$ for the single photon. The high-order PB phase will be kept in the relative phase between the $|H\rangle$ and $|V\rangle$ components of polarization as

$$|\psi_1'\rangle = \frac{1}{\sqrt{2}}\left(e^{j(m+\sigma)\Omega/2}|H\rangle + e^{-j(m+\sigma)\Omega/2}|V\rangle\right). \tag{S10}$$

To extract the geometric phase on the relative phase between the $|H\rangle$ and $|V\rangle$ components, we use a HWP at 22.5° to project $|\psi_1'\rangle$ into the basis of $|D\rangle = \frac{1}{\sqrt{2}}(|H\rangle + |V\rangle)$ and $|A\rangle = \frac{1}{\sqrt{2}}(|H\rangle - |V\rangle)$ as

$$\begin{aligned}
|\psi_1'\rangle &= \frac{1}{\sqrt{2}}\left[e^{j(m+\sigma)\Omega/2}a_D^\dagger + e^{-j(m+\sigma)\Omega/2}a_A^\dagger\right]|0\rangle \\
&= \frac{1}{2}\left[e^{j(m+\sigma)\Omega/2}(a_H^\dagger + a_V^\dagger) + e^{-j(m+\sigma)\Omega/2}(a_H^\dagger - a_V^\dagger)\right]|0\rangle \\
&= \frac{1}{2}\left[\left(e^{j(m+\sigma)\Omega/2} + e^{-j(m+\sigma)\Omega/2}\right)a_H^\dagger + \left(e^{j(m+\sigma)\Omega/2} - e^{-j(m+\sigma)\Omega/2}\right)a_V^\dagger\right]|0\rangle.
\end{aligned} \tag{S11}$$

Then, we retain only the $|H\rangle$ photons by using a PBS. The coincidence count between D1 and D2 is proportional to $|(e^{j(m+\sigma)\Omega/2} + e^{-j(m+\sigma)\Omega/2})|^2 \propto \{1 + \cos[(m+\sigma)\Omega]\}$. With the solid angle of

$\Omega = 4\theta$ on the high-order PS, the high-order PB phase can be calculated from the coincidence count $1 + \cos[4(m + \sigma)(\theta + \theta_0)]$, where θ_0 is the initial angle of the first set of DP+HWP. In experiment, we set $\theta_0 = \pi/4(m + \sigma)$ to get the minimum coincidence count at the beginning of the measurement and the coincidence count is proportional to $1 - \cos[4(m + \sigma)\theta]$.

III. High-order PB phase for N-photon Fock state

For a single photon with the initial state $|\psi_1\rangle$, undergo a cyclic transformation on the high-order PS converted into new states and return to its initial state $|\psi_1'\rangle$. It will acquire an high-order PB phase given by

$$|\psi_1'\rangle = e^{j\arg\langle\psi_1'|\psi_1\rangle}|\psi_1\rangle = e^{-j(m+\sigma)\Omega/2}a^{\dagger}|0\rangle, \tag{S12}$$

where $|0\rangle$ indicates vacuum state and $a^{\dagger}$ is the creation operator of photon. While, the N identical photons in the initial state $|\Psi_1\rangle$ undergo a cyclic transformation and also return to its initial state $|\Psi_1'\rangle$. N photons can be expressed as the Nth tensor product and the high-order PB phase for the evolution of an N-photon Fock state can be written as

$$|\Psi_1'\rangle^{\otimes N} = e^{jN\arg\langle\Psi_1'|\Psi_1\rangle}|\Psi_1\rangle^{\otimes N} = \frac{e^{-jN(m+\sigma)\Omega/2}}{\sqrt{N!}}(a^{\dagger})^N|0\rangle, \tag{S13}$$

where $\otimes$ represents the tensor product. So, N-photon Fock state are expected to acquire N times the geometric phase for one photon, showing the same result as the dynamic phase.

IV. Preparation and phase measurement of the N00N state

A linearly polarized beam at 390 nm pumps the type-II PPKTP2 to create a pair of collinear photons via the SPDC process. The PPKTP2 is kept at the phase-matching temperature of 25.6°C at the degenerate wavelength of 780 nm. After compensating the birefringent walk-off effect, the degenerate two photons with $|H\rangle$ and $|V\rangle$ can be written under the basis of $|R\rangle$ and $|L\rangle$ as

$$\begin{aligned} a_H^{\dagger}a_V^{\dagger}|0\rangle &= \frac{1}{\sqrt{2}}(a_R^{\dagger} + a_L^{\dagger})|0\rangle \otimes \frac{-j}{\sqrt{2}}(a_R^{\dagger} - a_L^{\dagger})|0\rangle \\ &= -\frac{j}{2}\left[(a_R^{\dagger})^2 - (a_L^{\dagger})^2\right]|0\rangle \\ &= -\frac{j}{2}\left(|2\rangle_R|0\rangle_L + |0\rangle_R|2\rangle_L\right). \end{aligned} \tag{S14}$$

The degenerate two photons become the N00N state in the basis of $|R\rangle$ and $|L\rangle$. After passing through the QWP at 45°, it will be transformed into the N00N state in the basis of $|H\rangle$ and $|V\rangle$

$$|\Psi_1\rangle = \frac{1}{\sqrt{2}}\left(|2\rangle_H|0\rangle_V + |0\rangle_H|2\rangle_V\right). \tag{S15}$$

For the initial state $|\Psi_1\rangle$, it undergoes a cyclic transformation and also returns to its initial state $|\Psi_1'\rangle$. A PB phase of $(m+\sigma)\Omega/2$ will be acquired for the $|H\rangle$ state and $-(m+\sigma)\Omega/2$ will be acquired for the $|V\rangle$ state for the single photon state. For the N00N state with $N = 2$, the high-order PB phase will be kept in the relative phase between the $|H\rangle$ and $|V\rangle$ components as

$$|\Psi_1'\rangle = \frac{1}{\sqrt{2}}\left(e^{j(m+\sigma)\Omega}|2\rangle_H|0\rangle_V + e^{-j(m+\sigma)\Omega}|0\rangle_H|2\rangle_V\right), \tag{S16}$$

which acquires 2 times the geometric phase compared with the single photon state.

To extract the geometric phase from the relative phase between the $|H\rangle$ and $|V\rangle$ components, we use a HWP at 22.5° to project $|\Psi_1'\rangle$ into the basis of $|A\rangle$ and $|D\rangle$ as

$$\begin{aligned}|\Psi_1'\rangle &= \frac{1}{2}\left[e^{j(m+\sigma)\Omega}(a_D^\dagger)^2 + e^{-j(m+\sigma)\Omega}(a_A^\dagger)^2\right]|0\rangle \\ &= \frac{1}{4}\left[e^{j(m+\sigma)\Omega}(a_H^\dagger + a_V^\dagger)^2 + e^{-j(m+\sigma)\Omega}(a_H^\dagger - a_V^\dagger)^2\right]|0\rangle \\ &= \frac{1}{4}\Big[(e^{j(m+\sigma)\Omega} + e^{-j(m+\sigma)\Omega})(a_H^\dagger)^2 + 2(e^{j(m+\sigma)\Omega} - e^{-j(m+\sigma)\Omega})a_H^\dagger a_V^\dagger \\ &\quad +(e^{j(m+\sigma)\Omega} + e^{-j(m+\sigma)\Omega})(a_V^\dagger)^2\Big]|0\rangle.\end{aligned} \tag{S17}$$

Then, we only retain the $|H\rangle$ two-photons by using a PBS and use a 1×2 fiber BS (50:50) to detect the two-photons. The coincidence count between D1 and D2 is proportional to $\left|e^{j(m+\sigma)\Omega} + e^{-j(m+\sigma)\Omega}\right|^2 \propto 1 + \cos[2(m+\sigma)\Omega]$. With the solid angle of $\Omega = 4\theta$ on the high-order PS, the high-order PB phase can be calculated from the coincidence count $1 + \cos[8(m+\sigma)(\theta+\theta_0)]$, where θ_0 is the initial angle of first DP+HWP. In experiment, we set $\theta_0 = \pi/8(m+\sigma)$ to get the minimum coincidence count at the beginning of the measurement and the coincidence count is proportional to $1 - \cos[8(m+\sigma)\theta]$.

V. Quantum Fisher information

As quantum PB phase evolves faster with the increase of number of photons in a state, one application could be super-sensitive measurement. For example, the relative rotation angle θ between two sets of DP+HWP. This prospect can be investigated by calculating the quantum Fisher information carried by a probe state $|\psi(\Omega)\rangle$ about a parameter Ω, which has the form as [47]

$$F_Q(|\psi(\Omega)\rangle) = 4\Delta\hat{H} \text{ with } \hat{H} = j\frac{d\hat{U}^\dagger(\Omega)}{d\Omega}\hat{U}(\Omega), \tag{S18}$$

where $\hat{U}$ is the unitary evolution of the state on the parameter space. $\hat{H}$ is the generator of the unitary $\hat{U}$ and its variance $\Delta\hat{H} = \langle\hat{H}^2\rangle_\psi - \langle\hat{H}\rangle_\psi^2$ is taken with respect to the input state. In our experiment, the unitary evolution $\hat{U}$ for the geometric phase acquired by the initial state can be written as

$$\hat{U} = e^{j\hat{N}(m+\sigma)\Omega\hat{\sigma}_z/2}, \tag{S19}$$

where $\hat{\sigma}_z$ is the Pauli matrix in the basis of $|H\rangle$ and $|V\rangle$ and $\hat{N}$ is the occupation operator of photon. The quantum Fisher information can be rewritten as

$$F_Q(|\psi(\Omega)\rangle) = \langle\psi(\Omega)|[\hat{N}^2(m+\sigma)^2 - 2\hat{N}(m+\sigma)\hat{\sigma}_z]|\psi(\Omega)\rangle. \tag{S20}$$

For the single photon state in $|\psi_1\rangle = \frac{1}{\sqrt{2}}(|H\rangle + |V\rangle)$, the quantum Fisher information can be calculated as $(m+\sigma)^2$. While, for the two-photon in N00N state $|\Psi_1\rangle = \frac{1}{\sqrt{2}}(|2\rangle_H|0\rangle_V + |0\rangle_H|2\rangle_V)$, the quantum Fisher information can be calculated as $4(m+\sigma)^2$. As the N00N state with N photons, the estimated quantum Fisher information $F_Q = N^2(m+\sigma)^2$.

The inversion of Fisher information gives a limit of variation of the measured parameter Ω, known as the Cramer-Rao bound. It satisfies $\delta^2(\Omega) \geq 1/MF(\Omega)$, where M is the times of measurement and $\delta(\Omega)$ is the standard deviation. Here, the lower bound of the standard deviation for measuring the geometric phase is proportional to $1/[N(m+\sigma)]$. The lower bound of $\delta(\Omega)$ decreases in proportion to $1/N$, which is also known as the Heisenberg limit. Hence, Measuring for N00N states with larger N should be able to enhance the sensitivity of quantum PB phase. Increasing the topological charge m can also effectively enhance the sensitivity.

In our experiment, our measurement method can achieve the minimum standard deviation predicted by quantum Fisher information (Heisenberg limit). To support our claim, we also calculate the classical Fisher information in our measurement method in experiment for the N00N state. According to Eq. (S17), the probability of the measurement result in the basis of $\{|x_i\rangle\} = \{|2_H, 0_V\rangle, |0_H, 2_V\rangle, |1_H, 1_V\rangle\}$ is

$$\begin{aligned} P_{|2_H,0_V\rangle} &= P_{|0_H,2_V\rangle} = \frac{1}{4}\{1+\cos[2(m+\sigma)\Omega]\}, \\ P_{|1_H,1_V\rangle} &= \frac{1}{2}\{1-\cos[2(m+\sigma)\Omega]\}. \end{aligned} \tag{S21}$$

Taking these probabilities into the definition of classical Fisher information [47]

$$F_{cl}(\Omega) = \sum_{x_i} \frac{1}{P(x_i|\Omega)} \left[\frac{\partial P(x_i|\Omega)}{\partial \Omega}\right]^2 = 4(m+\sigma)^2 = F_Q, \tag{S22}$$

which shows the same result as the definition of quantum Fisher information in the N00N state of $N = 2$. It shows that our measurement of the geometric phase is the optimal measurement, which reaches almost the Heisenberg limit.

VI. Supplementary experimental measurement results

The supplementary figure for comparing the geometric phases of the mode in $(m, \sigma) = (+2, 0)$ between the single-photon state and the N00N state, as shown in Fig. S2.

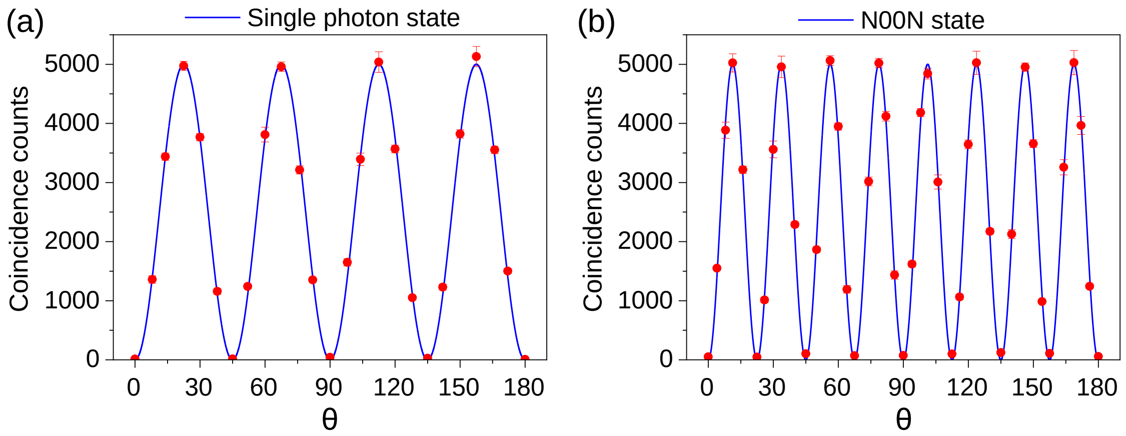

FIG. S2: Comparison of the geometric phases of the mode in $(m, \sigma) = (+2, 0)$ between the single-photon state (a) and the N00N state with $N = 2$ (b). The theoretical (blue solid line) and experimental (red dots) coincidence counts as a function of relative rotation angle θ between two DPs.
